# Comparison of Different Blood Collection, Sample Matrix, and Immunoassay Methods in a Prenatal Screening Setting

**DOI:** 10.1155/2014/509821

**Published:** 2014-07-15

**Authors:** Jeroen L. A. Pennings, Jacqueline E. Siljee, Sandra Imholz, Sylwia Kuc, Annemieke de Vries, Peter C. J. I. Schielen, Wendy Rodenburg

**Affiliations:** ^1^Centre for Health Protection (GZB), National Institute for Public Health and the Environment (RIVM), P.O. Box 1, 3720 BA Bilthoven, The Netherlands; ^2^Centre for Infectious Diseases Research, Diagnostics and Screening (IDS), National Institute for Public Health and the Environment (RIVM), P.O. Box 1, 3720 BA Bilthoven, The Netherlands; ^3^Department of Obstetrics, Wilhelmina Children's Hospital, University Medical Centre Utrecht (UMCU), P.O. Box 85090, 3508 AB Utrecht, The Netherlands

## Abstract

We compared how measurements of pregnancy-associated plasma protein A (PAPP-A) and the free beta subunit of human chorionic gonadotropin (f*β*-hCG) in maternal blood are influenced by different methods for blood collection, sample matrix, and immunoassay platform. Serum and dried blood spots (DBS) were obtained by venipuncture and by finger prick of 19 pregnant women. PAPP-A and f*β*-hCG from serum and from DBS were measured by conventional indirect immunoassay on an AutoDELFIA platform and by antibody microarray. We compared methods based on the recoveries for both markers as well as marker levels correlations across samples. All method comparisons showed high correlations for both marker concentrations. Recovery levels of PAPP-A from DBS were 30% lower, while those of f*β*-hCG from DBS were 50% higher compared to conventional venipuncture serum. The recoveries were not affected by blood collection or immunoassay method. The high correlation coefficients for both markers indicate that DBS from finger prick can be used reliably in a prenatal screening setting, as a less costly and minimally invasive alternative for venipuncture serum, with great logistical advantages. Additionally, the use of antibody arrays will allow for extending the number of first trimester screening markers on maternal and fetal health.

## 1. Introduction

Traditionally, screening for Down syndrome in The Netherlands is comprised of a combined biochemical and ultrasound test in the first trimester. Concentrations of pregnancy-associated plasma protein A (PAPP-A) and chorionic gonadotropin (f*β*-hCG) are measured in serum, while nuchal translucency thickness is measured by sonography. The values are normalized for gestational age as the multiple of the median (MoM) in the unaffected population. Usually, whole blood is collected by venipuncture and spun down to prepare serum at the collection location, where serum is stored at 4°C until shipped by mail.

Both f*β*-hCG and PAPP-A are suitably stable for routine screening, provided blood is promptly separated, serum is stored at refrigerator temperature, and transport times are kept to a minimum. Detected f*β*-hCG levels increase with storage and transport duration, and this effect is exacerbated by increases in temperature [1]. f*β*-hCG stability is greatly improved in dried blood spots compared to serum storage, while PAPP-A levels remain stable [2]. So, potentially, dried blood spots (DBS) are a superior sample matrix as compared to serum. Furthermore, DBS collection allows for using finger prick blood, which is a minimally invasive alternative for venipuncture serum and allows collection outside a laboratory, for example, at home or during a midwife visit. Both issues, finger prick collection and DBS sample matrix, have great logistical advantages, especially in a screening setting with a low population density and insufficient local availability of laboratory equipment. Thus, we set out to compare serum samples and dried blood spot samples, collected either by venipuncture or by finger prick, to establish whether obtained concentration measurements of PAPP-A and f*β*-hCG were comparable between the collection and sample matrix methods.

Furthermore, a number of additional parameters were recently discovered to potentially improve first trimester screening for aneuploidies (candidates are, for example, PlGF, ADAM12, AFP, PP13, and Inhibin A) [3–7] but also allow screening for adverse pregnancy outcome. It is well known that PAPP-A, PlGF, and PP13 are screening markers for preeclampsia, intrauterine growth restriction (IUGR), and intrauterine fetal demise [8–12]. Also, parameters for prenatal screening for irregular erythrocyte antigens and infectious diseases during pregnancies are preferably measured in the first trimester.

Current test methods require additional serum for each marker, although alternatively methods were recently introduced that allow multiple marker testing in small volumes in the range of finger prick serum or DBS volumes. An antibody-microarray is suited to potentially harbor dozens of biomarker tests within the same assay [13]. In a previous paper, we showed that there is good correlation between venous serum PAPP-A and f*β*-hCG concentrations measured with the AutoDELFIA analyzer and the antibody array [14]. In this study, we investigated whether the small amount of sample from finger prick and DBS collection in combination with an antibody array immunoassay platform is suitable for first trimester screening.

## 2. Methods

### 2.1. Study Design

Nineteen pregnant women in their first pregnancy trimester were asked to participate in this experiment as part of a larger study concerning the longitudinal changes of marker levels in blood. This study approach was approved by the Scientific Ethical Committee of the University Medical Centre Utrecht (METC Utrecht), The Netherlands (protocol number: 07-222). All participating women in this paper have given written informed consent to participate in this longitudinal study.

### 2.2. Blood Sampling

Sample collection took place between May and October of 2011. Blood was collected by venipuncture (5 mL) and by finger stick (0.5 mL) from all individuals. To obtain blood by finger stick, hands were briefly warmed up under lukewarm water and cleaned with alcohol, after which a finger stick needle was used to obtain blood. Blood was collected into a microtube without applying pressure to the finger. Directly after blood collection, a few drops of 50 *μ*L whole blood obtained by both methods were spotted onto a filter paper card (Ahlstrom 226, Ahlstrom Corporation, Helsinki, Finland) with a plastic pipette to generate spots of 1 cm in diameter that were allowed to air-dry for 3 hours at room temperature (RT). For serum preparation, blood samples were left to coagulate for 15–30 min at RT and centrifuged at RT for 10 minutes at 2000 g. Serum samples and dried filter paper cards were stored at −80°C until analysis.

### 2.3. AutoDELFIA Analysis

Analysis of PAPP-A and f*β*-hCG was performed in a single run with a 1235 AutoDELFIA analyzer, using an automated dissociation-enhanced lanthanide fluorescent immunoassay (AutoDELFIA; PerkinElmer, Turku, Finland). To analyze PAPP-A and f*β*-hCG in DBS, one 3 mm spot was punched into 96-well plates manually. Analysis was done according to the manufacturer's guidelines. Briefly, proteins were eluted for 3 hours in 150 *μ*L of buffer in wells precoated with anti-PAPP-A and f*β*-hCG antibodies. After incubation, buffer and paper filter disks were aspirated and wells were washed six times. PAPP-A and f*β*-hCG concentrations were quantified using Europium (Eu) and Samarium (Sm) labeled tracer antibodies. After dissociation of tracer antibodies and label and chelation of the Eu and Sm ions, the amount of Eu and Sm label was measured by excitation with a 340 nm laser and detection of emitted light at 610 and 650 nm, respectively.

### 2.4. Antibody Array Production

Capture antibodies mouse anti-human PAPP-A 10E1 (Hytest, Turku, Finland) and mouse anti-human hCG Beta 7 (Acris Antibodies GmbH, Herford, Germany) were diluted in 2x Protein Array Buffer (Whatman, Kent, UK) to a concentration of 1 mg/mL for anti-PAPP-A and 0.5 mg/mL for anti-f*β*-hCG. Antibodies were spotted on 16-array nitrocellulose FAST-slides (Whatman) using a Piezorray Noncontact Microarraying System (PerkinElmer, Wellesley, MA, USA). Two drops per position, of an estimated 330 pL/drop, were spotted under humidity below 40%. Four replicates of each antibody were arrayed to ensure adequate statistics. The slides were stored in a desiccator cabinet (Nalgene, Rochester, NY, USA). Standards for PAPP-A and f*β*-hCG were obtained from the AutoDELFIA kits. These standards were calibrated against the WHO International Reference Preparation (PAPP-A: 78/610 for SP1, f*β*-hCG: 75/551).

### 2.5. Antibody Array Analysis

To analyse PAPP-A and f*β*-hCG in DBS, two 6.35 mm (1/4 inch) disks were punched into a tube with 120 *μ*L PBS, 0.5% Tween-20 (Surfact Amps 20, Thermo Fisher Scientific, Rockford, USA). Disks were eluted for 45 minutes during shaking at 500 rpm. Filter disks with eluate were transferred to Swab Extraction Tube System (Roche Applied Science, Germany) and centrifuged for 10 min at 2200 g. Arrays were blocked in 100 *μ*L Protein Array Blocking Buffer (Whatman) and subsequently incubated with 90 *μ*L of 1 : 10 diluted serum, eluted DBS, or pooled PAPP-A and f*β*-hCG standards. Arrays were washed (PBS, pH 7.4, 0.05% Tween, Sigma-Aldrich) and incubated with a mix of biotinylated goat anti-human PAPP-A (1 : 100, R&D Systems, Minneapolis, MN, USA) and biotinylated mouse anti-human hCG 28A4 (1 : 250, Hytest) detection antibodies. After incubation with diluted DyLight649-conjugated Streptavidin (1 : 500, Jackson ImmunoResearch Laboratories, West Grove, PA, USA) arrays were washed with deionised water and were dried by vacuum.

### 2.6. Data Extraction, Data Analysis, and Statistical Analysis

Arrays were scanned with a Confocal Microarray Scanner (PerkinElmer) at a resolution of 10 *μ*m. ScanArray Express software V4.0 (PerkinElmer) was used to quantify the intensity of each spot using the adaptive circle method. Corrected median intensity values for each spot (median intensity minus local median background) were used for further analysis. Median intensity values of the four replicate spots were calculated using Microsoft Excel. Parameter fitting was performed with the statistical programme “*R*” (http://www.r-project.org/), based on the parameter logistic log (4PL) model *Y*(*x*) = *D* + (*A* − *D*)/(1 + (*x*/*C*)^*B*^) [15].

Comparison of concentrations was done using Passing-Bablok regression. *R* values > 0.90 were considered to indicate similarity of concentrations.

## 3. Results

### 3.1. Population Characteristics

The current markers PAPP-A and f*β*-hCG concentrations in serum and dried whole blood spots, obtained from 19 singleton pregnancies, were measured and compared. Median maternal age was 33 years (range: 25–41), median maternal weight was 70 kg (range: 57–110 kg), and median gestational age was 88 days (range: 76–97 days) (1 sample in week 10, 1 in week 11, 12 in week 12, and 5 in week 13).

### 3.2. Collection: Finger versus Vein Serum

**Figure 1:**
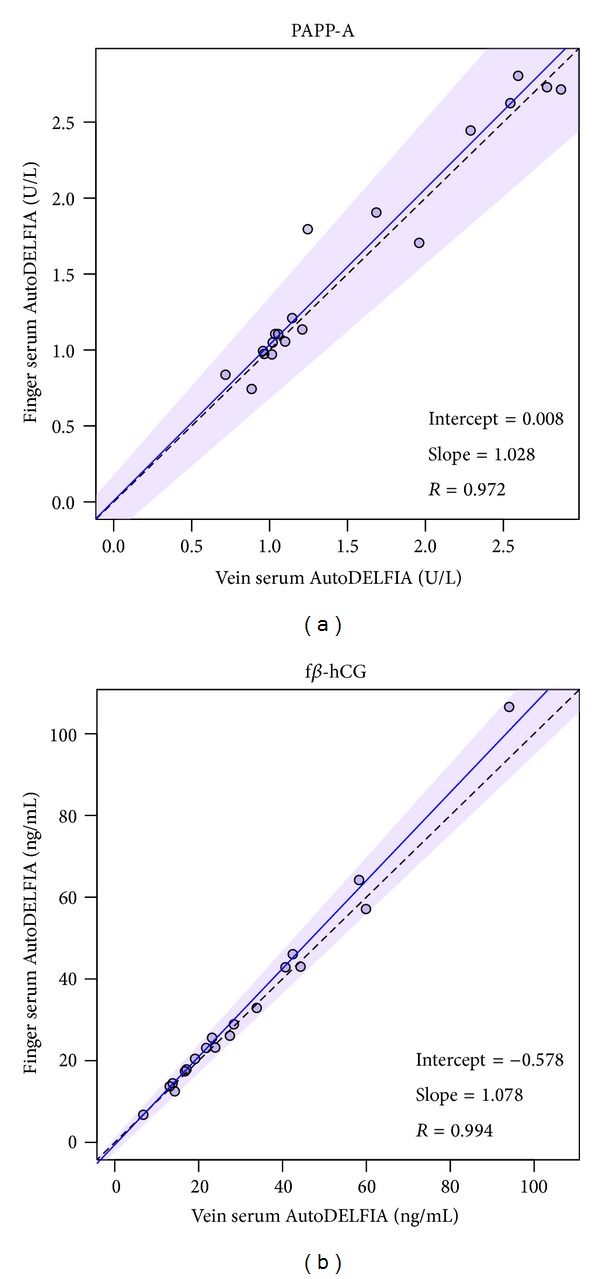
Comparison blood collection method. Passing-Bablok regression was performed on finger versus vein serum samples for PAPP-A (a) and f*β*-hCG (b). Solid line, regression line; shaded area, 95% confidence interval; dashed line, identity line.

Serum collection from either vein or finger does not affect PAPP-A and f*β*-hCG concentrations (Figure 1). The correlations for PAPP-A and f*β*-hCG were both *R* > 0.97 (Figures 1(a) and 1(b), resp.) and the average recoveries were 103% (Table 1).

### 3.3. Sample Matrix: Serum versus DBS

**Figure 2:**
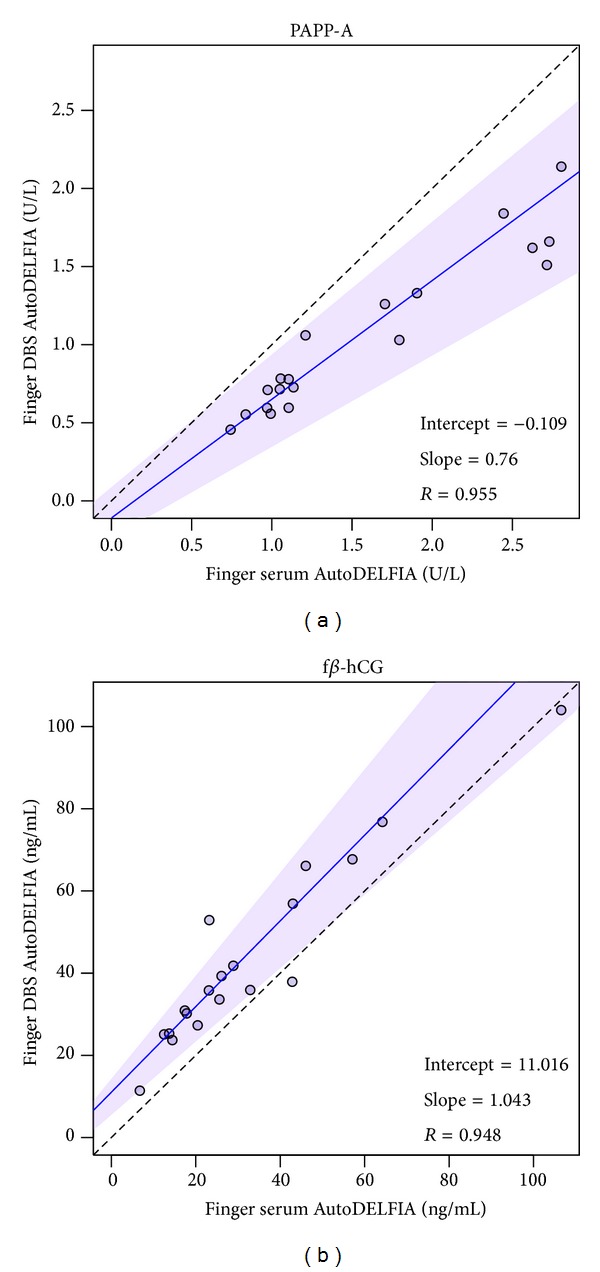
Comparison sample matrix. Passing-Bablok regression was performed on finger DBS versus finger serum for PAPP-A (a) and f*β*-hCG (b). Solid line, regression line; shaded area, 95% confidence interval; dashed line, identity line.

f*β*-hCG recovery is increased in DBS compared to serum storage, corresponding to previous data [16]. This is independent of venous (156%) or finger origin (151%) (Table 1). PAPP-A shows less recovery from a DBS sample matrix for both venous and finger prick blood, showing recoveries of 71% in venous DBS and 67% in finger DBS (Table 1). Correlations between finger DBS and finger serum were >0.94 for both markers (Figure 2). Moreover, comparisons across both blood collection methods as well as sample matrices showed similar quantitative performance across all four conditions, with *R* > 0.9 for PAPP-A (see Supplementary Figure 1 in Supplementary Material available online at http://dx.doi.org/10.1155/2014/509821) as well as f*β*-hCG (Supplementary Figure 2). To summarize, the results show that both finger and vein collection and serum and DBS matrix are allowed for both markers.

### 3.4. Antibody Array Platform

An antibody array platform allows parallel analysis of multiple markers, with the performance of the array being similar to AutoDELFIA, as shown in our previous paper [14]. In this study, to examine the feasibility of using antibody arrays in a screening assay, we first set out to corroborate the quantitative performance shown for AutoDELFIA earlier in this paper, namely, across sample collection and sample matrix, when the corresponding samples are analysed on antibody array platform. Correlations between finger serum versus vein serum and finger DBS versus finger serum, respectively, were always >0.9 when measured on antibody array platform (Supplementary Figure 3). Moreover, these correlation values were similar to those determined using AutoDELFIA. The slopes for the latter comparison were decreased for PAPP-A and increased for f*β*-hCG. This, too, corresponds to differences in recovery found using the AutoDELFIA platform (Table 1).

### 3.5. Antibody Array versus AutoDELFIA

**Figure 3:**
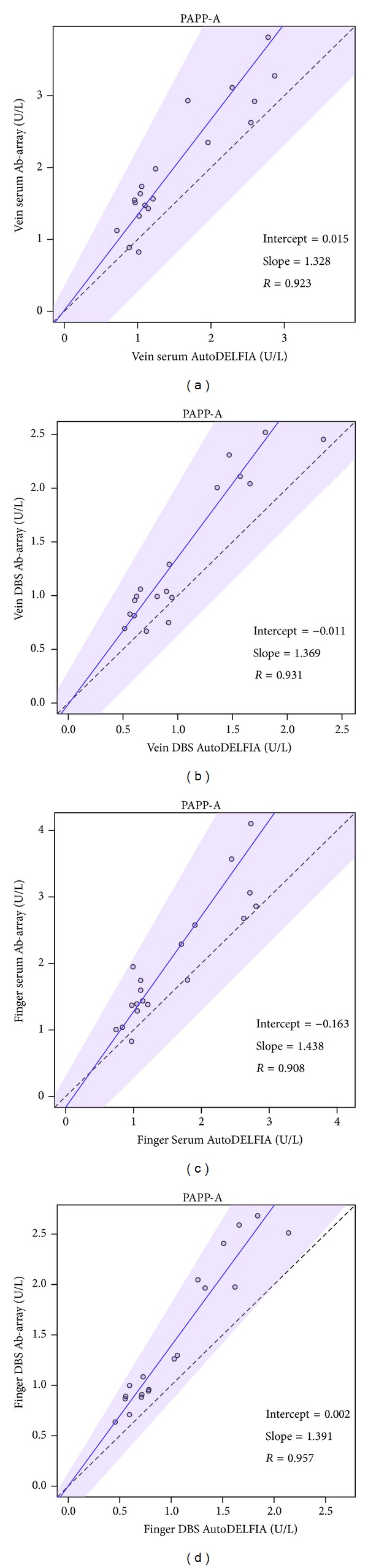
Antibody array versus AutoDELFIA performance for PAPP-A. (a) Vein serum. (b) Vein DBS. (c) Finger serum. (d) Finger DBS. Solid line, Passing-Bablok regression line; shaded area, 95% confidence interval; dashed line, identity line.

**Figure 4:**
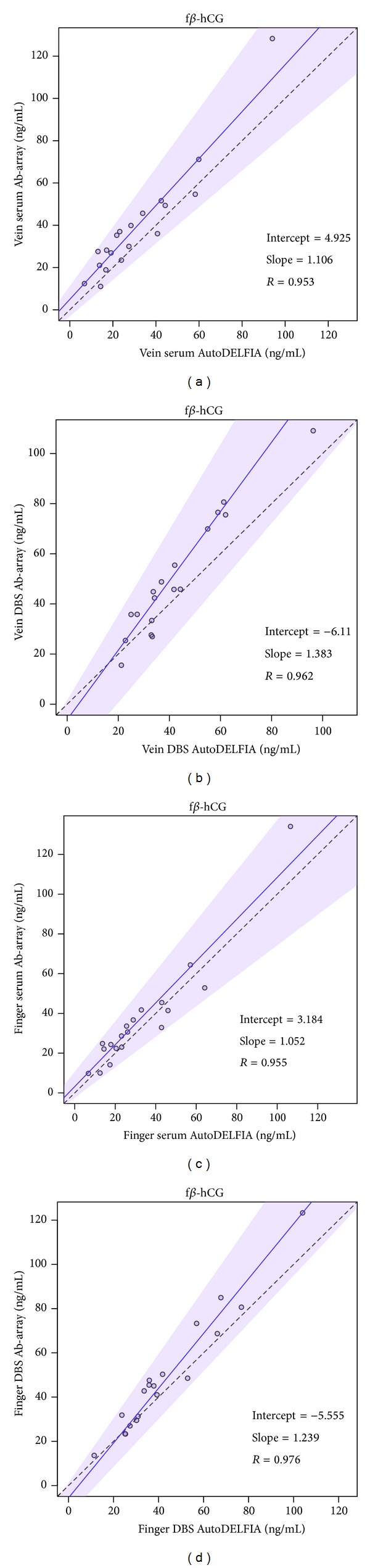
Antibody array versus AutoDELFIA performance for f*β*-hCG. (a) Vein serum. (b) Vein DBS. (c) Finger serum. (d) Finger DBS. Solid line, Passing-Bablok regression line; shaded area, 95% confidence interval; dashed line, identity line.

In addition to testing consistent performance on an antibody array (i.e., within a platform), we expanded the comparison of AutoDELFIA versus antibody array (i.e., across platforms) to the four blood collection and sample matrix methods mentioned above (vein-serum, vein-DBS, finger-serum, finger-DBS). Using current samples on two platforms, we find that the four pairwise comparisons give consistent correlation *R* values > 0.9 (Figures 3 and 4).

**Figure 5:**
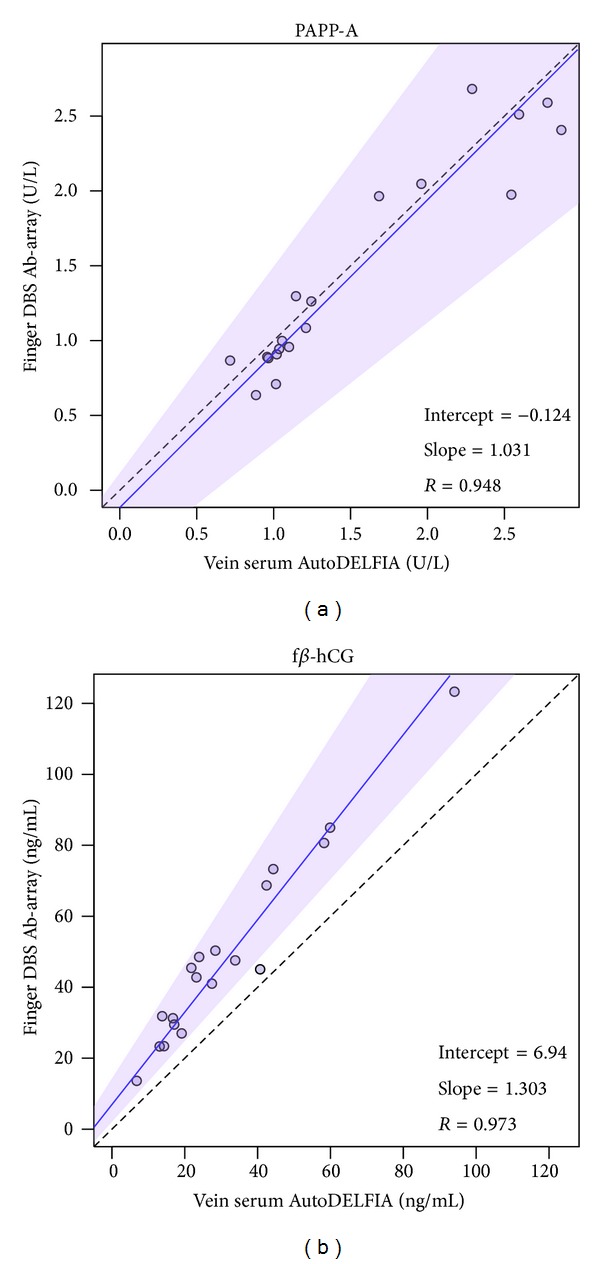
Finger DBS antibody array versus vein serum AutoDELFIA, for PAPP-A (a) and f*β*-hCG (b). Solid line, Passing-Bablok regression line; shaded area, 95% confidence interval; dashed line, identity line.

Interestingly, we also found that the comparison between finger serum antibody array and vein serum AutoDELFIA yielded correlation *R* values > 0.9 for both PAPP-A and f*β*-hCG (Figure 5). This indicates that a combined change in blood collection, sample matrix, and immunoassay platform does not compromise overall screening assay performance.

Ultimately, finger DBS would allow improved logistics including extension of the number of biomarkers when used in combination with an array platform. Finger DBS in combination with an array platform showed high correlations with the DELFIA platform using currently used venous serum samples (Figure 5). The average recovery of PAPP-A and f*β*-hCG in finger DBS compared to conventional venipuncture serum recovery is summarized in Table 1.

## 4. Discussion

The purpose of this study was to compare two methods for blood collection (venipuncture and finger prick) and two sample matrices (serum and DBS) that can be used in prenatal screening. Furthermore, we compared two immunoassay platforms (AutoDELFIA and antibody array) to characterize the performance of a potential multimarker platform for future prenatal screening purposes in the first trimester. A particular aim of this work was to perform multiple method comparisons in parallel, so that not only pairwise methodology comparisons are possible, but we can also give a proof-of-principle for the feasibility of multiple simultaneous methodological changes in a prenatal screening setting, such as moving from conventional venous serum analysis by AutoDELFIA to finger prick obtained DBS analysis by antibody array (Figure 5).

Regarding blood collection, we show here that concentrations of PAPP-A and f*β*-hCG in serum from venous sampling are comparable to those in serum from finger prick blood.

The choice of samples matrix affects both protein recovery and stability upon storage. Regarding the latter, it has been shown by Cowans et al. [2] that f*β*-hCG stability is greatly improved in DBS compared to serum storage. As for protein recovery, we show that this is similar for venous and finger prick collection, but the recoveries in dried blood spots are about 45% higher. PAPP-A recovery is 30% lower in DBS than in blood, independent of venous or finger origin. Different recovery or amounts in DBS compared to serum are probably due to the fact that measurements in DBS are actually measurements in blood and not in serum. This difference between DBS and serum was described earlier and has been attributed to f*β*-hCG subunit release by dissociation of hCG occurring faster in blood than in serum [2, 16]. On the other hand, similar dissociation of PAPP-A from the heterotetrameric complex with MBP would lead to conformation changes and a decreased detection by assay antibodies [2]. To cope with this in screening is to prepare separate mathematical equations for the relationship between gestational age and median concentrations measured in blood spots [17] and to adapt risk calculation algorithms to take into account differences in population distribution parameters [2, 16]. Thus, as a sample matrix, serum and DBS are suitable (provided calculations appropriate for the laboratory protocol are applied) with DBS offering logistical and storage stability advantages.

Thirdly, we show that those concentrations measured with the conventional AutoDELFIA technique are comparable to those measured with the antibody array assay. The current conventional venous serum AutoDELFIA test showed high correlation with the finger DBS determination on arrays for the current Down syndrome markers.

For all of the comparisons made in this study, we found high correlations with linearity between concentration measurements extending across a concentration range representative of first trimester prenatal screening.

Taken together, the findings of this paper release a plethora of possibilities for screening using biochemical markers in the first trimester. Instead of a venous puncture, a finger prick can be used to obtain either serum or a DBS. Instead of a 4.5 mL tube, only a microvial will suffice to mail the sample to the laboratory. Finger prick in combination with DBS would also be less costly in sampling and transportation. A DBS would even give the additional advantage of far superior preservation in transport. Antibody arrays allow extension of marker sets using DBS (or small blood volumes), for example, to combine DS testing with other trisomies or other adverse pregnancy outcomes.

Wider implementation of the methodologies envisaged in this proof-of-principle paper would, however, require validation using more samples and multilaboratory comparisons. These would serve to determine adapted mathematical relations between marker levels and gestational age (based on the assay methodology as well as other laboratory factors such as ambient temperature), as well as to assess quantitative performance.

## 5. Conclusion

In conclusion, we show that consistent quantitative performance of PAPP-A and f*β*-hCG measurements across blood collection, sample matrix, and immunoassay platform allows for improved sample logistics and screening tests.

## Supplementary Material

Supplementary figure 1: Comparison of blood collection (vein and finger) and sample matrix (serum and DBS) values for PAPP-A, using AutoDELFIA.
Supplementary figure 2: Comparison of blood collection (vein and finger) and sample matrix (serum and DBS) values for f*β*-hCG, using AutoDELFIA.
Supplementary figure 3: Effects of blood collection and sample matrix using an antibody array platform. Finger serum versus vein serum for PAPP-A (A) and f*β*- hCG (B); finger DBS versus finger serum for PAPP-A (C) and f*β*-hCG (D).

## Figures and Tables

**Table 1:** Average recovery percentage of dried blood spots and finger stick versus conventional venipuncture serum of PAPP-A and f*β*-hCG for both immunoassay platforms.

Average % recovery compared to venipuncture serum (± SD)				
Platform	Marker	Serum finger prick	DBS venous	DBS finger prick
AutoDELFIA	PAPP-A	103 ± 13	71 ± 12	67 ± 9
Ab-array	PAPP-A	100 ± 10	70 ± 12	72 ± 11
AutoDELFIA	f*β*-hCG	103 ± 7	156 ± 56	151 ± 32
Ab-array	f*β*-hCG	90 ± 8	137 ± 38	132 ± 34
