# Supplementary figures and images for: Comparison of Different Blood Collection, Sample Matrix, and Immunoassay Methods in a Prenatal Screening Setting
Source: Dis Markers. 2014 Jul 15;2014:509821. doi: 10.1155/2014/509821 (PMC4123521; doi:10.1155/2014/509821)

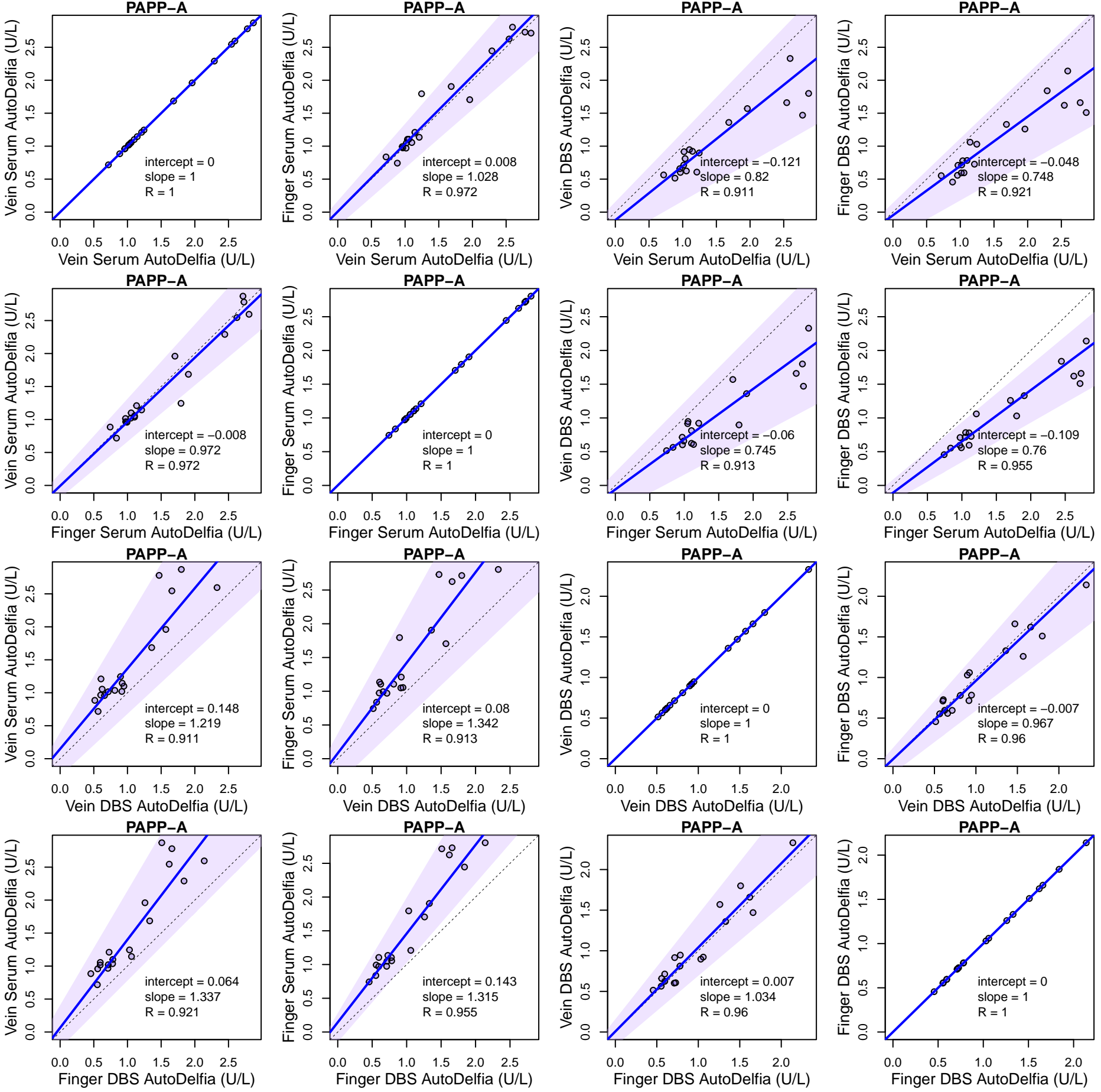

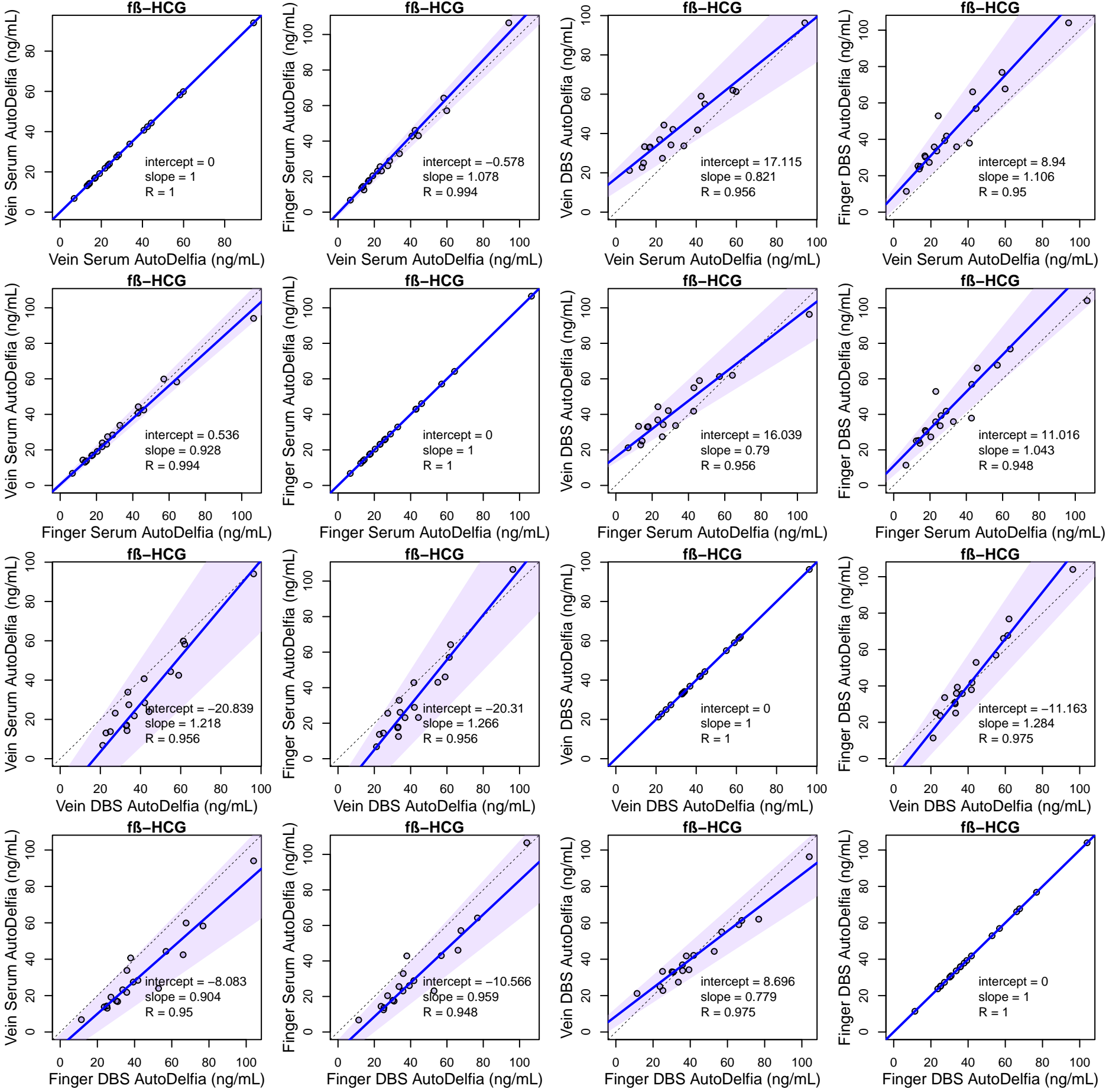

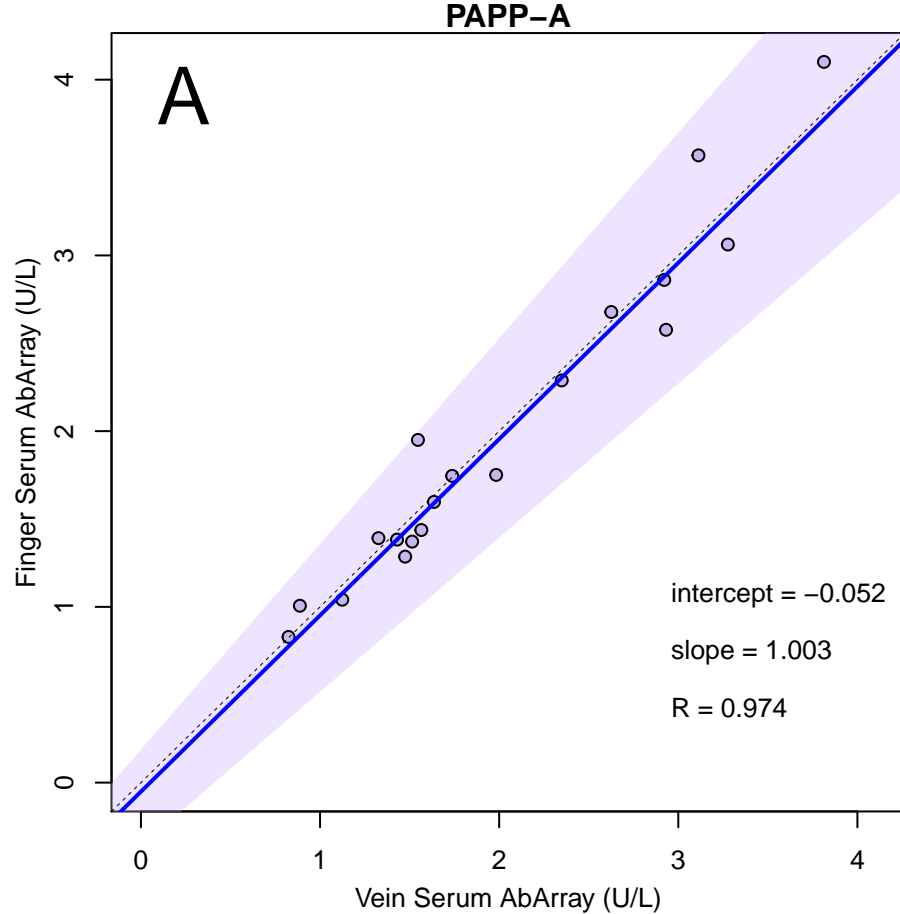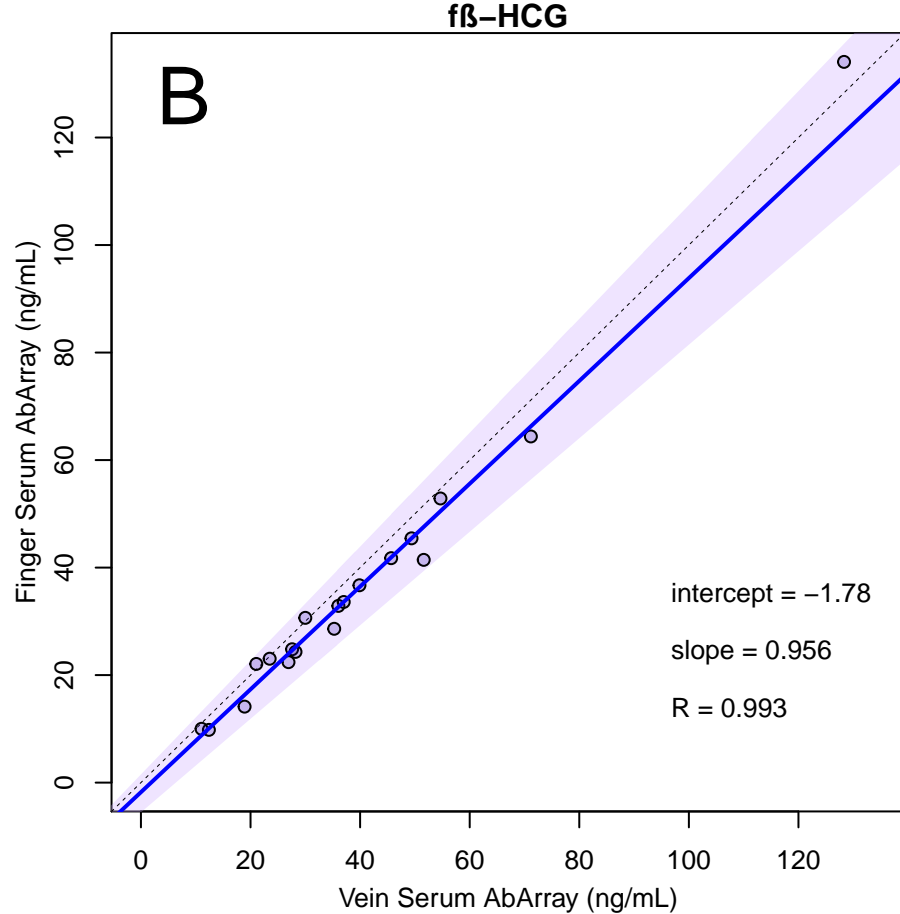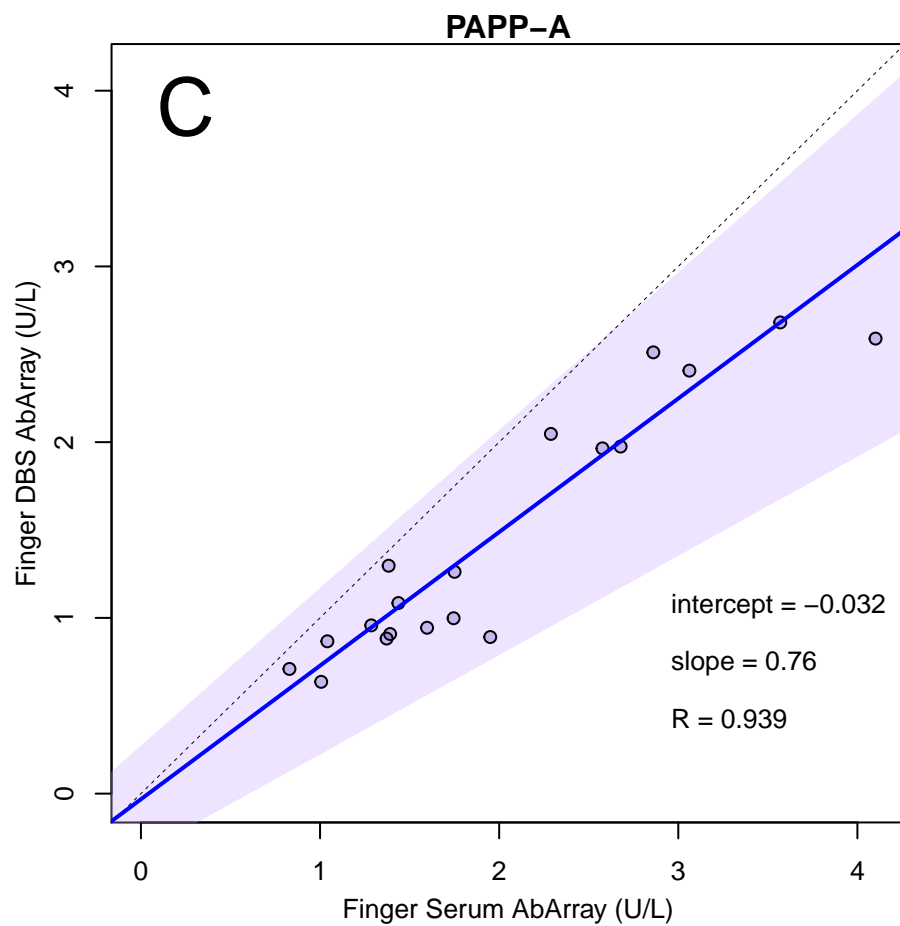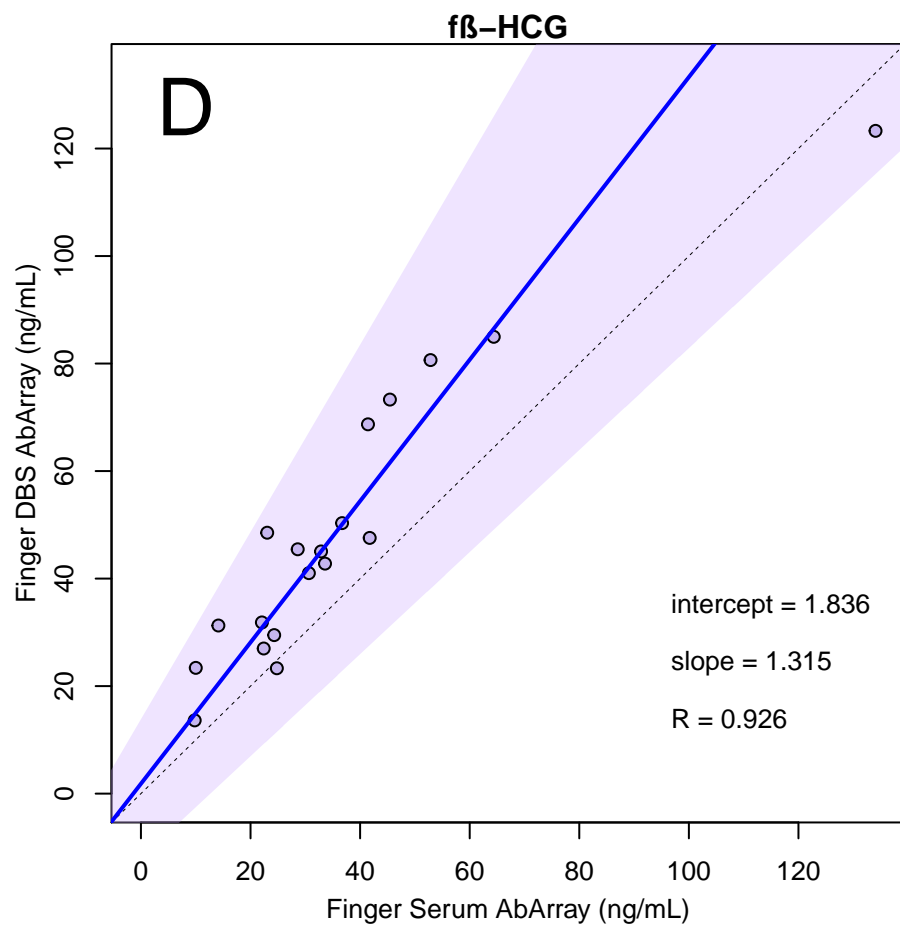

Supplement: Supplementary file 1 — Supplementary figure 1: Comparison of blood collection (vein and finger) and sample matrix (serum and DBS) values for PAPP-A, using AutoDELFIA. Supplementary figure 2: Comparison of blood collection (vein and finger) and sample matrix (serum and DBS) values for fβ-hCG, using AutoDELFIA. Supplementary figure 3: Effects of blood collection and sample matrix using an antibody array platform. Finger serum versus vein serum for PAPP-A (A) and fβ- hCG (B); finger DBS versus finger serum for PAPP-A (C) and fβ-hCG (D). [file 509821.f1.pdf]
